# Evaluation of a pharmacy supported e-cigarette smoking cessation intervention in Northwest England

**DOI:** 10.1186/s12889-022-13711-x

**Published:** 2022-07-12

**Authors:** Alan D. Price, Margaret Coffey, Lawrence Houston, Penny A. Cook

**Affiliations:** School of Health and Society, University of Salford, Salford, UK

**Keywords:** Smoking cessation, Electronic cigarettes, E-cigarettes, Pharmacy, Mixed-methods, Longitudinal, Community intervention

## Abstract

**Background:**

Cigarette smoking cessation has been described as the world’s most important public health intervention. Electronic cigarettes are a relatively new tool for assisting smoking cessation but there is a lack of data on their efficacy. This article reports on a pharmacy supported e-cigarette smoking cessation intervention undertaken in a metropolitan area in the north of England.

**Methods:**

Longitudinal mixed-methods evaluation incorporating analysis of secondary data, interviews with service users, and interviews with service providers at 3-month and 12-month follow-up, with an additional text message survey of service users at 12-month follow-up.

**Results:**

The four-week follow-up data suggest that for every twenty people given an e-cigarette, six quit smoking tobacco and three people cut their cigarette intake by more than five cigarettes per day. Long-term follow-up results were positive but only a small number of participants were still engaged with the study at 12 months. Service users and providers spoke positively about the combination of e-cigarettes and pharmacy support.

**Conclusions:**

E-cigarette distribution combined with pharmacy support appears to be an agreeable and effective intervention for smoking cessation, but further data are needed on long-term quit rates and health effects.

## Background

Smoking cessation has been described as potentially the most important public health intervention globally, if effective [1]. It can offer several health and financial benefits to smokers and their families [2, 3], including the reduction of second-hand smoke exposure [3]. However, despite many smokers reporting a desire to stop smoking [4], successful, long-term cessation is challenging. Barriers include: stress and smoking as stress management; the social acceptability of smoking especially in low-income and marginalised communities; and a lack of effective interventions, particularly those involving contact with healthcare professionals [5]. Due in part to such barriers, rates of quitting smoking may be as low as 3%, and rates of relapse may be as high as 80% [6].

Individual-level interventions to promote smoking cessation include brief interventions, behavioural interventions, nicotine replacement therapy (NRT), pharmaceutical therapies, and combinations of these such as a behavioural intervention combined with pharmaceutical treatment [1, 7, 8]. Brief interventions take the form of a short conversation between a healthcare practitioner and their patient or client, often lasting only a few seconds or minutes [7]. There is evidence of their effectiveness, albeit at fairly low levels, but this is seen as an efficient use of time [9]. Behavioural interventions typically consist of regular support meetings with a healthcare professional, and will often be combined with another form of intervention such as NRT. They can be delivered to individuals or groups over a number of weeks and may involve some form of recognised therapy such as cognitive behavioural therapy [7]. A recent systematic review found stronger evidence of efficacy for brief interventions compared to longer-term behavioural interventions [10]. The use of local pharmacies in delivering smoking cessation services has been shown to increase the efficacy of programmes [11]. Pharmacies represent a cost-effective and efficient system of delivering evidence-based interventions alongside practitioner advice and support [11].

Electronic cigarettes (e-cigarettes) are small, hand-held, battery-operated devices that resemble cigarettes. They work by heating a solution containing nicotine which is then inhaled as an aerosol into the lungs of the user (known as vaping) [12]. As well as representing an effective method of nicotine delivery, they may also be beneficial as an aid to smoking cessation due to their hand-to-mouth and inhalation actions, and the presence of a visible vapour released upon exhalation, resembling the experience of smoking [1]. E-cigarette users (known as vapers) in the UK have reported enjoying the experience of using their e- cigarettes, and were able to switch easily from conventional cigarettes, especially due to their effective nicotine delivery and the action of vaping as similar to that of cigarette smoking [13]. A Cochrane systematic review, first published in 2014 and most recently updated in 2021, found moderate evidence that e-cigarettes outperform NRT and behavioural support. The same review found no evidence of serious adverse events linked to e-cigarettes, only non-serious adverse events such as headache, throat irritation, cough and nausea [14]. There is some debate around the safety of e-cigarettes and long-term data are still needed [15], but some studies have concluded that the risks associated with vaping are minimal when compared to the well-known health risks associated with smoking conventional cigarettes, and that e-cigarettes are preferable [16–18]. The UK Royal College of Physicians has endorsed the use of e-cigarettes as an aid to smoking cessation, and Public Health England have reported that e-cigarettes are around 95% less harmful than conventional cigarettes [15]. Recently, e-cigarettes have been added to the list of recommended smoking cessation interventions in England by the National Institute of Health and Care Excellence [19]. Around 6% of adults in the UK report using e-cigarettes, compared to around 14–15% who smoke tobacco [20]. Between 40% and 50% of vapers are thought to also smoke tobacco. Vaping is becoming more popular than nicotine replacement therapy and it seems to be increasingly seen as a long-term solution, with around 40% of vapers reporting their use at over three years in 2020, compared to a little over 20% in 2018 [20]. However, public opinion is mixed on the safety of e-cigarettes, with 15% believing them to be more harmful than smoking, 29% believing that vaping is safer, and 38% believing the two to be equally harmful [20].

In 2017, the Greater Manchester Health and Social Care Partnership (GMHSCP) [21] published a strategy to reduce adult smoking in the city region to 13%, or by 115,000 smokers. As part of that plan, two local authorities in Greater Manchester introduced e-cigarette pilot schemes delivered by pharmacies. In 2018, the first of these schemes, an evaluation of which has already been published [22], recruited 1022 smokers, 383 (37%) of whom had quit smoking tobacco after four weeks. Among those still smoking tobacco, the average number of cigarettes smoked per day had dropped from 19.1 to 8.7. People in less deprived areas and working in higher-paid jobs were more likely to have quit tobacco. The study concluded that such schemes appear to be effective, but more work was needed on targeting smokers from lower income backgrounds, and interventions needed to be followed up over a longer period. Here we report on the second pilot scheme, which used similar methods to the first programme: a pharmacy-supported smoking cessation intervention using e-cigarettes, which targeted smokers in manual and routine occupations. This current evaluation additionally included a 12 month follow up.

## Methods

### Intervention

Between January and June 2019, a pharmacy-supported smoking cessation pilot scheme (funded by the local authority) was delivered in a metropolitan borough in Greater Manchester by six pharmacies. The aim of the project was to offer smoking cessation support and free e-cigarettes, chargers and liquid to around 800 residents who were routine and manual workers, as well as social housing tenants. Potential clients for the service were recruited by a press release, workplace marketing and posters in social housing facilities and by word of mouth. Attendance was incentivised by free equipment and refills. Pharmacies were chosen that had existing skills in delivering smoking cessation services. Service users (*n* = 871) were provided with e-cigarettes, a charger, and fluids. All equipment was sourced from a partner e-cigarette supplier and provided by pharmacies, who also gave practical advice and support on smoking cessation and e-cigarette use, which was available throughout the programme.

### Design

The evaluation team were not involved in the design of the scheme, nor in the recruitment of service users. This report describes an analysis of routinely collected service use data which were collected by the pharmacies (secondary data), and interviews conducted by the research team (primary data). The evaluation consisted of a longitudinal mixed-methods evaluation, with phase one data collected from zero to three months, followed by a 12-month follow-up at phase two. The evaluation was designed to:

- Measure smoking abstinence and/or smoking reduction across 12 months
- Explore the impact and perceived value of the project on current smokers’ behaviour
- Explore the experience of delivering the project from a pharmacy perspective, including facilitators and barriers to encouraging participants to stop smoking using an e-cigarette
- Understand the experience of engaging in the project, including facilitators and barriers to quitting smoking using an e-cigarette

#### Primary and secondary data sources and procedure

##### Phase 1

**Fig. 1:**
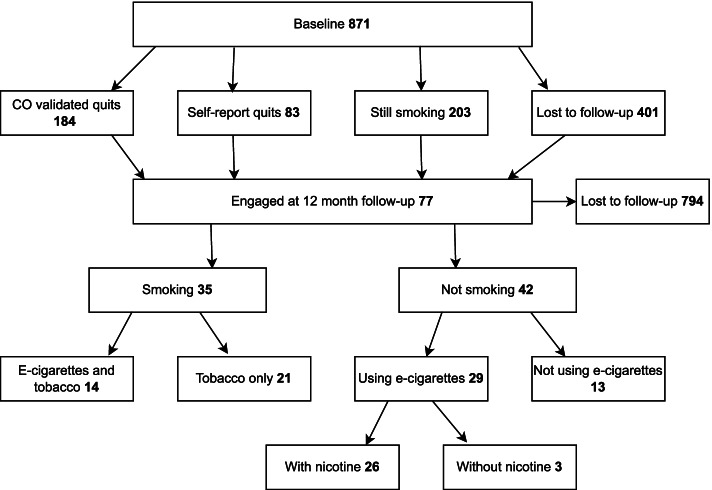
Flow chart of pharmacy clients through the first and second stages

Figure 1 shows a flow chart of pharmacy clients through both stages of data collection. Service users (*n* = 871) were enrolled in the scheme for three months. Routinely collected data were taken from service users at baseline, 2 weeks and 4 weeks (endpoint). Service users were also asked if they would be happy to be contacted about their experience, and those consenting gave a contact telephone number. Routine data were collected by pharmacies on service user demographics (including age, gender, occupation and Index of Multiple Deprivation (IMD), previous use of e-cigarettes, smoking status, carbon monoxide (CO) readings (to corroborate self-reported smoking status at baseline and four weeks), and data related to e-cigarette fluid provision (e.g. flavour and strengths).

After three months, service users were interviewed by the research team (*n* = 26), to discuss their participation in the project, especially in terms of the approach, the impact of the intervention on their smoking behaviour, and any barriers or facilitators to quitting cigarettes. Recruitment to interviews was discontinued when thematic saturation was reached. Interviews were conducted face-to-face or by telephone. Interviews were held with a member of staff from five out of the six service providers (*n* = 5), to discuss their experience of delivering the scheme, approaches that were taken to recruitment and delivery, the effectiveness of those approaches, their perceived impact on smoking behaviour, and barriers and facilitators to encouraging smokers to quit smoking using an e-cigarette. One service provider declined to take part in the interviews.

##### Phase 2

**Fig. 2:**
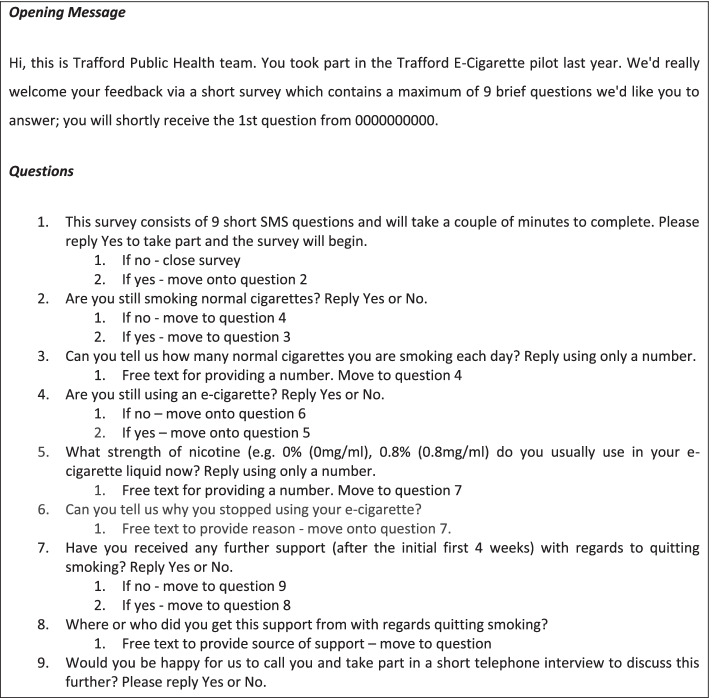
Text message survey options (phase 2)

After 12 months, the borough council sent text messages to all service users with a valid phone number on record and who had provided consent to be contacted. Secondary analysis of the call records revealed that a total of 466 text messages were sent; 355 were successfully delivered and 77 service users returned surveys. As shown in Fig. 2, service users were asked about their current use of cigarettes and e-cigarettes, the strength of nicotine used in e-cigarettes, and about any support they were receiving. They were also asked whether they would consent to an interview. The details of those consenting for interview were passed to the research team. Fifteen participants were asked about their cigarette and e-cigarette use since the intervention, their experience of the intervention and the support they received from the service providers, and about any other sources of support post-intervention. Interviews were also held with service providers (*n* = 4), who were asked about their experience of the intervention, any service users who had kept in touch, perceived barriers and facilitators to quitting or reducing cigarette smoking, and their thoughts on potential future interventions. Two service providers declined to take part in the interviews.

### Analysis

Quantitative data were analysed descriptively. Characteristics of quitters were compared to those who did not quit using chi square tests. Qualitative data were analysed thematically, using the methods recommended by Braun and Clarke [23].

## Results

### Phase 1 (3-month evaluation): secondary data

As shown in Fig. 1, a total of 871 service users were provided with e-cigarettes and advice from pharmacies involved in the intervention. Service users were invited to present back to the pharmacy after four weeks, and those who did not attend were contacted by SMS (text message). At this stage, 401 service users were unresponsive and lost to phase one follow-up. Of the remaining 470 service users, 184 (21.1% of those originally enrolled) were confirmed as having quit smoking tobacco via self-report along with a negative carbon monoxide reading (less than five parts per million). A further 83 (9.5%) service users reported that they had stopped smoking tobacco, but either did not present for a CO test or were CO positive when tested (greater than five parts per million). A total of 203 (23.3%) service users reported that they had not stopped smoking tobacco at this stage.

Table 1 shows a comparison between those who were and were not smoking tobacco at four-week follow-up. Confirmed and unconfirmed quitters are grouped together (quit), and those who were lost to follow-up were assumed to be still smoking tobacco and are grouped together with the self-reported smokers (not quit). The overall quit rate was 267 (30.7%). There was no significant difference in quit rate based on age or gender. Service users who identified as white (27.8%) were less likely to have quit than those who identified as non-white or preferred not to state their ethnicity (44.6%). There was significant quit variation between occupational groups; those in the managerial and professional occupations were most likely to quit (40.0%), while those who were unemployed were the least likely (20.5%). Similarly, quit rates were the lowest in the most deprived quintile (25%) and highest in the least deprived (40%). However, it should be noted that, due to the targeting of the intervention in the areas of most need, the absolute numbers of quitters was higher in the most deprived quintile (73 individuals) than in the two most affluent quintiles (24 individuals). Finally, there was also a significant effect of baseline smoking category, where the lightest smokers were most likely to quit (37%) and the heaviest smokers least likely (26%).

**Table 1:** Comparison of baseline characteristics of those who quit tobacco at four-week follow-up with those still using tobacco (or lost to follow-up*) at four-week follow-up (phase 1)

	Quit N (%)	Not quit N (%)	Total	Chi	df	***P***
**Gender**						
Female	140 (28.6)	349 (71.4)	489	0.70	1	0.402
Male	116 (31.3)	255 (68.7)	371			
**Age group**						
18–24	17 (21.3)	63 (78.8)	80	4.76	5	0.446
25–34	51 (27.3)	136 (72.7)	187			
35–44	47 (31.5)	102 (68.5)	149			
45–54	70 (32.9)	143 (67.1)	213			
55–64	49 (30.1)	114 (69.9)	163			
65+	22 (32.4)	46 (67.6)	68			
**Ethnicity**						
White	211 (27.8)	548 (72.2)	759	12.0	1	0.001
Non-white/prefer not to say	45 (44.6)	56 (55.4)	101			
**Occupational status**						
1 Unemployed	54 (20.5)	210 (79.5)	264	21.6	6	0.001
2 Home Carer	12 (24.5)	37 (75.5)	49			
3 Managerial and Professional	40 (40.0)	60 (60.0)	100			
4 Intermediate	34 (37.4)	57 (62.6)	91			
5 Routine and Manual	68 (34.2)	131 (65.8)	199			
6 Retired	29 (33.0)	59 (67.0)	88			
7 Sick or Disabled	19 (27.5)	50 (72.5)	69			
**IMD Quintile**						
Most deprived	73 (24.9)	220 (75.1)	293	11.3	4	0.023
2	53 (26.8)	145 (73.2)	198			
3	68 (36.6)	118 (63.4)	186			
4	34 (30.6)	77 (69.4)	111			
Least deprived	24 (40.0)	36 (60.0)	60			
**Baseline category of smoking**						
Lowest (1–10)	72 (36.5)	125 (63.5)	197	8.1	2	0.018
Medium (11–19)	78 (32.5)	162 (67.5)	240			
Highest (20+)	102 (25.8)	294 (74.2)	396			
Missing	4	23	27			
**Total**	256 (29.8)	604 (70.2)	860			

Importantly, even those who reported still smoking tobacco (‘non quits’) recorded having halved the number of cigarettes smoked (from 19.3 to 8.7 cigarettes per day, *n* = 178) and halved the level of CO recorded (15.4 to 8.6 ppm, *n* = 104). Sixty-one percent of the ‘non-quitters’ had reduced their cigarette consumption by more than 5 cigarettes a day.

### Phase 1 interviews with service users

The evaluation found six key themes in relation to service users’ experiences of taking part in the e-cigarette project. Supporting quotes are shown in Table 2.

**Table 2:** Themes and supporting quotes for phase 1 service user interviews

Theme	Example supporting quotes
Quality of service provided	“Well it was excellent, they rang me when I needed to go in for a check you know and to refill the liquid, because they gave them free of charge and they did a CO2 reading, to check the smoke in the blood, in the lungs and what have you, and he was very, very nice the man that was there to see me.” (Female; dual-use*).
Health and financial benefits of e-cigarette use	“I think it’s a very good idea, I really do. Yeah, let’s put it like this, at the time, my chest and my breathing was really, really, really bad, from smoking cigarettes, even though I didn’t smoke that many, and then I started on the e-cigarette, I still haven’t cut them out 100%, I’ve cut them out about 95%, but this e-cigarette it’s just like, it’s saved my life if you like.” (Male; dual-use). “Yeah definitely and it’s not only saved me money, from not buying cigarettes, but it’s saved me money from initially getting into my vaping. You know because it was a free service so.” (Male; dual use*).
Effectiveness of e-cigarette	“Well it takes the craving away definitely. I believe really, it’s just because you’re using your hands and your kind of imitating smoking.” (Male; quit).
Mental health	“I started to use it but then, unfortunately my mum got poorly and has passed away so, I don’t think it’s been the right time for me to completely … I probably wouldn’t be smoking, I would have carried on using it, but because of my circumstances, my mind frame wasn’t in it.” (Female: smoker).
Beginning the quit journey using an e-cigarette	“When I first started with it, I was choking a lot, where obviously I wasn’t used to it, but once you get used to it, it’s basically a simple object to use. But it’s just trying to get used to it all.” (Female, dual-use*)
Concerns over e-cigarette use	“It’s, technically you’re burning oil and inhaling the vapours off oil, so that surely, that’s got to have some sort of effect on your lungs, or in the long term you know?” (Male; dual-use*).

* dual use = smoking tobacco and vaping

#### Quality of service provided

Service users reported a high level of satisfaction in the service they received from the providers. Many discussed receiving useful, detailed advice about the e-cigarettes and how to use them. This seems to have been a key facilitator in helping to maintain the effective use of e-cigarettes. They also reported high levels of satisfaction with the quality of the devices, which were seen as superior to devices that some service users had tried in the past. Higher quality of e-cigarettes was also seen as conducive to maintaining e-cigarette use and staying off tobacco. For many participants, the devices used in this study were seen as otherwise difficult to afford, so the intervention was an opportunity to step up to that higher level of quality. Participants also noted that the scheme was well advertised; they had heard about it from multiple sources including word of mouth, from a GP or nurse, an advertisement on social media or in a local newspaper, or at their local pharmacy.

#### Health and financial benefits of e-cigarette use

Participants described health and financial benefits of switching from tobacco to e-cigarettes. Use of the devices along with cutting down on tobacco was seen as having a noticeable improvement on health, especially in terms of respiratory problems. This was seen as leading to improvements in everyday life. In terms of the financial benefits, participants reported economic savings from buying less tobacco, and in terms of the devices being free as part of the scheme, which was gratefully accepted. A combination of health and financial benefits was seen as being a major incentive to use e-cigarettes instead of tobacco.

#### Effectiveness of e-cigarettes

Participants largely reported that the e-cigarettes were an effective tool for helping to reduce, and in some cases stop, tobacco use. Some noted that the hand-to-mouth action that resembles cigarette smoking was a useful mechanism. Criticisms were minor, but some participants noted that the resemblance was not perfect – they contrasted the ‘hit’ from smoking tobacco with the less noticeable effect of vaping.

#### Mental health

Mental health and tobacco smoking were seen as closely related. When participants were experiencing times of stress, they found it more difficult to stop smoking tobacco, even when vaping was an option. Stress or anxiety were described as triggers that led to increased tobacco smoking or decreased interest in stopping. This was sometimes seen as temporary - for example, when experiencing a bereavement, one participant described how she felt it was not the right time to stop smoking.

#### Beginning the quit journey using an e-cigarette

Participants described an ‘adjustment phase’, where some time was needed to get used to the feel of using the e-cigarettes. This was partly to do with finding a pleasing flavour and strength of liquid. There was a certain amount of trial and error involved, and it sometimes took a few different combinations until the participant were happy with the outcome. There was also an adjustment to the feel of the e-cigarette, which was different from smoking tobacco. Some participants reported side-effects such as coughing or that the sensation of inhaling vapour was unpleasant at first. However, these issues were described as temporary. Two participants also reported technical problems with their devices which rendered them unusable.

#### Concerns over e-cigarette use

Many participants expressed concerns over potential health impacts of vaping. The inhalation of vapour into the lungs was seen as harmful. The lack of certainty around any long-term impacts was concerning to some. Although they recognised the well-established risks of smoking tobacco, the unknown risks associated with vaping were worrying.

### Phase 1 interviews with service providers

Service providers described their experience of delivering the project; its impact on participants’ smoking behaviour; programme facilitators and barriers for participants stopping smoking using the e-cigarette; and personal facilitators and barriers for quitting smoking. Supporting quotes are shown in Table 3.

**Table 3:** Themes and supporting quotes for phase 1 service provider interviews

Theme	Example supporting quotes
Experience of delivering the project	“People are more excited about this one and we’ve seen a lot of people who’ve been smoking for years who’ve actually given up, which makes us feel like we’re helping” (Pharmacy 5)
Pharmacist perceptions of the impact of the e-cigarette intervention	“… we’ve got quite a lot of people that have given up and they’ve still given up, you know even though it might be two or three months later down the line … so for those people obviously a huge impact because you had people smoking you know 40 cigarettes a day” (Pharmacy 3).
Programme facilitators to delivering the e-cig intervention	“There was advertising. They were advertising in the local paper, we have advertising in store and on our windows and also in the local surgery … and I believe it went in the Manchester Evening News website as well” (Pharmacy 1).
Programme barriers to delivering the intervention	I think there’s been a spectrum of impact, I think there’s been quite a few people who have just got the e-cig because it’s free and not really you know tried it to some extent, that … weren’t really invested and they’ve not done typically well” (Pharmacy 3).
Personal facilitators and barriers for quitting smoking	“I think sometimes it’s helpful if they’ve got demonstrable health problems that are linked to their smoking” (Pharmacy 3). “I think also the stress that they go through … life stress … we do have a lot of low-income people, I think … and it boils up, you know … they still want to have a cigarette when they’re going through the stress, instead of having another puff on the e-cig” (Pharmacist 1).

#### Pharmacists’ experience of delivering the intervention

Most of the pharmacists reported that the intervention was positive and effective. It was seen as successful in terms of helping people to stop smoking tobacco, and in some cases people who pharmacists felt would likely struggle, managed to quit. The success of the intervention was motivating for the pharmacists, who were keen for the scheme to continue. The use of an e-cigarette intervention specifically was seen as more effective than schemes using other tools, such as nicotine patches or gum. The key to this was the fact that vaping resembles tobacco smoking to a large extent.

#### Pharmacist perceptions of the impact of the e-cigarette intervention

Pharmacists described apparent age-related differences in the characteristics of participants. There was a feeling that younger participants were less likely to quit tobacco; even though they were using the e-cigarettes, the pharmacists reported that younger people were still smoking as well. Middle-aged and older service users were seen as more likely to quit tobacco. More broadly, pharmacists noted that the intervention was having positive effects on quitting, with even heavy and long-term smokers managing to cut down or quit. They estimated that around 50–70% of participants were managing to completely come off tobacco. Pharmacists described hearing about participants’ improved health, especially related to breathing and energy levels, and about the money reportedly saved. One group of participants were apparently planning to go on holiday using the money they had saved.

#### Programme facilitators to delivering the e-cig intervention

Pharmacists felt that the success of the scheme, in terms of reaching high numbers of people, was largely down to effective and widespread advertising. The formal scheme advertising conducted through newspapers and in pharmacies apparently continued to spread substantially by word of mouth. The fact that free e-cigarettes were being offered was popular with the public, so the nature of the scheme may have been the mechanism for continuing word-of-mouth spread. Another facilitator of success was that the intervention was conducted in a relaxed high-street setting rather than a more clinical doctors’ office or hospital. The pharmacy consultations were seen as highly productive; this was seen as an opportunity to give tailored advice to service users and support and encourage them during the quitting process. For example, some service users talked about still smoking the occasional cigarette and this was seen as a failure, but pharmacists were able to re-frame this as merely a lapse or 'a bump along the road' towards quitting. The e-cigarettes themselves were seen as helpful toward quitting, especially since they were seen as a new and advanced technology. This was especially true of the bigger Arc Slim variety, which had longer lasting batteries and adjustable strength. The use of a carbon monoxide monitor at the pharmacies was described as a useful way of motivating service users to quit. It was capable of both negative and positive reinforcement depending on the result. Finally, pharmacists reported being well-supported by the borough council in terms of responding to questions and replacing stock.

#### Programme barriers to delivering the intervention

Pharmacists highlighted a number of potential barriers to the success of the project, which were at least relevant to some participants. Barriers tended to fall into one of four categories: a lack of commitment from some service users; the short timeframe of the study; difficulties related to products and ordering; and a lack of referrals from primary healthcare providers.

Some service users seemed to lose interest after they were supplied with their equipment. Many service users were not able to be contacted after this point, with phone numbers that were out of service. This was especially common in people who were not previously known to the pharmacist. Whilst advertising of the scheme was successful at least in terms of numbers, pharmacists felt that some service users did not understand the nature of the intervention – people seemed to be aware of the e-cigarette giveaway but not the consultations and carbon monoxide testing. The four-week timeframe of the intervention was described as too short on two counts: one because it meant high demand by a large number or people over a short space of time, putting pressure on the staff; and two because four weeks was not seen as sufficient time for smokers to switch from tobacco to e-cigarettes. Finally, there were some difficulties with the availability and delivery of additional liquids to the pharmacies.

#### Personal facilitators and barriers for quitting smoking

Pharmacists were able to identify a number of personal (to the service users) facilitators and barriers to quitting tobacco. Service users were seen as more likely to cut down or quit if they used their e-cigarette regularly as a means of overcoming the urge to smoke. People who had existing health problems were seen as being more highly motivated to quit in order to improve their health. People who had other people around them who were involved in the programme and trying to quit were seen as more likely to succeed, with one pharmacist noting that some groups of men formed competitions with one another, and so had a competitive motivator to quitting. Service users with higher levels of stress in their lives were seen as less likely to quit smoking, as were those with other drug or alcohol dependence and mental health issues. Having access to cigarettes was also noted as a factor – if people were in an environment (at work or at home) with cigarettes available, they were seen as more likely to smoke.

### Phase 2 (12-month follow-up): text message survey

**Fig. 3:**
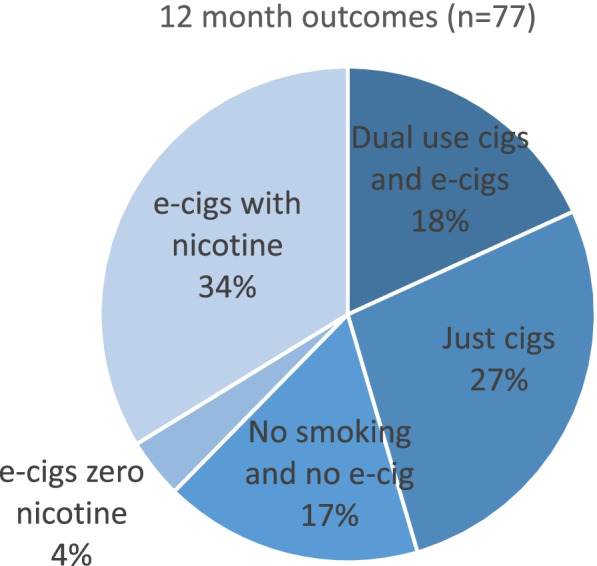
Outcome of text message survey at 12 months (phase 2)

Text messages were sent to service users at 12-month follow-up (see Fig. 2 for questions). A total of 335 messages were successfully delivered (phone numbers were available and in service). Of those successfully delivered, 77 service users returned a full survey. Over half of the retained service users reported that they had quit smoking tobacco (*n* = 42, 55% of those retained). Figure 3 shows the outcomes of all 77 service users. Those who had quit tobacco are shown in three categories: 17% of the sample (of 77) were not smoking and not using an e-cigarette (i.e. given up nicotine entirely); 4% were using e-cigarettes without nicotine; 34% were still using an e-cigarette with nicotine. Those who were still smoking tobacco were either using both tobacco and e-cigarettes (18%) or using tobacco only (27%).

Table 4 shows the 77 service users who were still engaged at 12-month follow-up, their status from phase one, and their status at phase two. There was significant variation between categories: the most likely group to have quit at 12 months were those who were confirmed (negative for carbon monoxide) to have quit at four weeks (23 quit, 3 not quit); those who were unconfirmed but self-reported as quit at 4 weeks were less likely to have quit, followed by those who were lost to follow-up at 4 weeks, and finally those who were still smoking at 4 weeks (*χ*^*2*^ = 23.18,*p* < 0.001). This significant difference had a large effect size (ϕ = .55, *p* < 0.001). Service users who used e-cigarettes as well as normal cigarettes reported smoking statistically significantly fewer cigarettes (8.7) than those who just smoked cigarettes (14.4) (*F* = 5.79, *df* = 1,33, *p* = 0.022).

**Table 4:** Service user status at twelve month outcome, compared to their status at four weeks (*n* = 77)

Four week outcome	Outcome at 12 months				Total
	Not quit		Self-reported quit		
	n	%	n	%	
Lost to follow-up	10	28.6	6	14.3	**16**
Not quit	17	48.6	5	11.9	**22**
Self-reported quit	5	14.3	8	19.0	**13**
CO-Confirmed quit	3	8.6	23	54.8	**26**
**Total**	**35**	**100**	**42**	**100**	**77**

Service users who were still smoking cigarettes, but not using e-cigarettes, were invited to submit a free-text response as to reasons why they were no longer using the device. The most common reason  given was problems with the equipment, including procuring and using it (*n* = 7). This was followed by not liking the taste (5), the e-cigarette breaking (4) and it causing physical symptoms, e.g. chapped lips or coughing (3). Other reasons for giving up e-cigarettes included bad press (1), poor quality of the product (1), and the expense (1). Among service users who were no longer using either cigarettes or e-cigarettes, the most common reason for no longer using their e-cigarettes was that they had used them to quit cigarettes and now no longer needed them (*n* = 5). Other reasons included not liking them (2), losing them (1), and claiming never to have had one (2).

### Phase 2 interviews with service users and providers at 12 months

As part of the 12-month evaluation, 15 interviews were held with service users and four with pharmacists. The participants spoke positively about the intervention, especially the support from pharmacists and the quality of the e-cigarettes. They also spoke about the connection between stress and smoking, and this related to the ongoing Covid-19 pandemic and associated restrictions. Pharmacists spoke about their satisfaction with the intervention and that they felt it raised the profile of smoking cessation. They felt that the use of pharmacies was a useful way to access people in the local community, and they also described some problems related to the devices and their supply to the pharmacies. For the purpose of this analysis, the focus will be on new or different themes from those that were identified in phase one.

#### Stress, smoking, and Covid-19

As described in the phase one analysis, participants reported that smoking was related to stress. During times of stress there is a greater need to smoke, or cravings are more difficult to ignore. For some, this meant switching back to tobacco from e-cigarettes, at least for a short time. Several participants reported successfully swapping to e-cigarettes for a considerable amount of time as a result of taking part in the intervention, but then relapsing and returning to smoking tobacco due to stress.

*“The fact that I was stressed out that’s why I went back to the cigs, because I did it really well for about 3 months, and then, I was under a lot of stress and then I just went back to cigs.”* (Female, Smoker)

During the period between the phase one and phase two analyses (approximately June 2019 to June 2020), there were considerable social changes in the UK, which were related to the Covid-19 pandemic. Colloquially referred to as ‘lockdown’, millions of people were asked to stay in their homes and were only allowed out for essential reasons. These altered living arrangements led to a documented increase in stress and reduced health and wellbeing generally [24]. The participants in this study spoke in depth about the Covid-19 pandemic having a negative effect on their smoking behaviour. Feedback from some participants described how lockdown posed additional challenges in their efforts to quitting smoking due to increased stress, worry and isolation. This resulted in some participants smoking more than usual while others stopped using their e-cigarettes altogether and returned to smoking cigarettes.

*“Well I am back smoking now, and I am smoking quite a lot and I am not liking it, I am really scared now I am smoking. I was ok for a bit and then lockdown happened and then just probably changes in life I would say, with what was happening. Stressing.”* (Female, Smoker)

#### Pharmacies as an effective community hub for smoking cessation interventions

Pharmacists reiterated the positive benefits of using their services as part of community health interventions, and especially for those involving smoking cessation. The combination of pharmacy advice and support along with free provision of the devices was seen as an effective strategy for smoking cessation. This specific intervention was seen as having raised the profile of smoking cessation in the local area, and especially in relation to e-cigarettes. Apparently there had been some negative impressions of e-cigarettes – some people saw them as dangerous and scary – which pharmacists were able to counter with evidence-based advice.

*“I think the other good thing from this... is that I think it...maybe raised the profile of e-cigs...because I think when they see it sort of coming from a pharmacy and from you know like the smoking clinic teams as well, it is kind of saying you know e-cigs are safe to use those. I think when they first came out they were sort of seen as maybe a bit dangerous weren’t they, and...some of the models that were out weren’t necessarily, they got a bit of bad press, so I think that has done good for e-cig in general yes. For raising its profile so, yes.”* (Pharmacy 1)

Pharmacists’ roles as primary healthcare providers within local communities puts them in a unique position to be able to give advice daily and locally to high numbers of local people. There was a strong sense of connection to the local community and pharmacists were keen to give as much help and support as possible to service users during their efforts to quit smoking. They reported that they wanted to create a sense of ‘togetherness’ between service provider and service user, to help build trusting relationships with participants and remove any barriers that may have prevented them from seeking help during the intervention.

*“Yeah I think they think pharmacy is a good idea to come to. To come and talk or just get some more advice from the pharmacist as well, whatever it is.”* (Pharmacy 4)

## Discussion

The aim of the pilot scheme was to encourage smokers to quit tobacco by switching to e-cigarettes with the support and advice of their local pharmacist. The aims of this evaluation were to use secondary data to assess the efficacy of the scheme in terms of number of quitters and reduced smoking among smokers, and to explore the experiences of service users and providers (pharmacists). Quantitative and qualitative assessments were commenced at three months (phase 1) and 12 month follow-up (phase 2). Both phases showed evidence that service users reduced their use of tobacco. A total of 871 people took part in the scheme, with 18% (confirmed by CO testing) to 26% (confirmed and unconfirmed) of the sample no longer using tobacco at 3 months, and 55% of those remaining in contact with the scheme, having quit tobacco at 12-month follow-up (5% of full sample). Service users and providers spoke positively about their experiences, while also providing useful descriptions of factors associated with the struggle of nicotine dependency.

Taken in context with the previously published Greater Manchester pharmacy-led e-cigarette intervention [22], these studies show that e-cigarettes appear to be an agreeable and effective alternative to tobacco, which can aid smoking cessation, especially when combined with pharmacy support. The qualitative findings in this report suggest that the pharmacy support was an important factor, and this may be largely due to the fact that pharmacies represent a bridge between primary care and the local community. Since the previous Greater Manchester pilot scheme concluded that longer-term follow-up was required, this study included assessments at both 3-months and 12-months. However, the majority of participants were not engaged with the scheme at 12-month follow-up, which diminished the power of the long-term quantitative assessment. Only 77 out of 871 (8.8%) participants provided any data at 12 months. Further studies should be designed to overcome this limitation, perhaps by conducting a 6-month follow-up or by offering an incentive.

In the wider context of smoking cessation interventions, these results are comparable in terms of the proportion of abstinent participants at follow-up. The most comprehensive review of e-cigarette interventions shows that the proportion of participants in similar studies (where the full cohort was supplied with e-cigarettes) who had stopped smoking at three-month follow-up was between 19% and 44%, while at 12-month follow-up the rate was between 14% and 53%. In the present study, 18% (confirmed) to 26% (confirmed and unconfirmed) had quit smoking at 3 months, and 55% of those who were still in contact had quit at 12-month follow-up (5% of full sample). Only one such study [25] followed up for longer than 12 months, where there was a decrease in abstinence over time. Further studies should be designed to follow up over longer periods of time, while longer-term and ongoing interventions may be helpful to counteract the tendency to relapse [14].

The longitudinal design and mixed-methods analysis of this data collected in a real-world setting represent strengths of this study, along with the fact that data were collected from service providers as well as service users. The findings provide insight into the practicalities, effectiveness and experience of such an intervention. However, this was an evaluation of a scheme which made use of routine service data, rather than an experimental study. We were therefore unable to measure potential useful modifiers or outcomes, such as tests of nicotine dependence or a measure of depression index. Similarly, we were unable to include a control group, the lack of which limits the ability to form reliable conclusions about the efficacy of the intervention. Confounding variables such as the placebo effect and regression to the mean may explain some of the reduction in smoking seen here, especially since there is a general trend toward smoking reduction in the UK [26]. Further intervention evaluations should compare e-cigarette provision plus support with at least one control group.

## Conclusion

Up to one quarter of people given a free e-cigarette and fluid, and advice on how to use it, stopped smoking tobacco in four weeks. A further 61% of those who were still smoking had reduced their intake by five cigarettes per day. It is difficult to draw firm conclusions from the phase two data due to low levels of participant engagement at that stage, but the experiences of both service users and providers over both phases was positive. Although more data are required on long-term health effects, e-cigarettes appear to be an agreeable and effective smoking cessation therapy and interventions similar to this one are recommended.

## Data Availability

The anonymised datasets used and/or analysed during the current study are available from the corresponding author on reasonable request.
